# Supplementary material for: Tumor cell-specific inhibition of MYC function using small molecule inhibitors of the HUWE1 ubiquitin ligase
Source: EMBO Mol Med. 2014 Sep 24;6(12):1525–41. doi: 10.15252/emmm.201403927 (PMC4287973; doi:10.15252/emmm.201403927)
Supplement: Supplementary file 1 — Supplementary Information [file emmm0006-1525-sd1.pdf]

Supplementary Information

Supplementary Figure S1: Proliferation of colorectal cancer cell lines upon depletion of HUWE1.

A. RQ-PCR documenting expression of *HUWE1* mRNA in the indicated cell lines expressing shRNA targeting *HUWE1*. Error bars display SD from technical triplicate assays from one representative experiment (n=2).

B. Colony formation assays of the indicated cells. Cells were plated sparsely and stained with crystal violet five days later.

Supplementary Figure S2: Growth of tumors in response to depletion of HUWE1.

A,B: Ls174T cells expressing doxycycline-inducible shRNAs targeting *HUWE1* were grafted subcutaneously into immunocompromised mice. Seven days after engraftment of the cells tumors became palpable and doxycycline treatment was started (1 mg/ml in drinking water). Tumor growth was followed for two weeks. (A) The upper panel shows data for individual mice for shHUWE1-2. The lower panel shows the average for this shRNA. p-values were calculated using Student's t-test (*p-value<0.05, **p-value<0.01). (B) Data shown for individual mice for shHUWE1-3. The average is shown in Figure 1E.

C. Ls174T cells expressing shHUWE1-3 were grafted orthotopically in the caecum of immunocompromised mice. Doxycycline treatment was started (1 mg/ml in drinking water) when mice showed an abdominal luciferase signal (14 days after engraftment) and tumor growth was followed for six weeks by measuring luciferase activity. Luciferase activity is shown for each individual mouse. * Mice were sacrificed. RLU: relative light units.

Supplementary Figure S3: Effect of depletion of HUWE1 on MYC target genes in wildtype and in p53-depleted cells.

A. Depletion of HUWE1 and p53 in Ls174T cells. Cells were stably infected with viruses expressing shHUWE1, shp53 or control as shown. After selection cells were harvested and immunoblots of cell lysates were probed with indicated antibodies.

B. RNA was isolated from the cells shown in panel (A) and gene expression was analyzed by RQ-PCR. Error bars show SD of triplicate technical assays from one representative experiment (n=2).

Supplementary Figure S4: Role of HUWE1 in DNA damage-induced degradation of MCL1.

A. UVB-induced degradation of MCL1 in HUWE1-depleted cells. U2OS cells were stably infected with retroviruses carrying either an shRNA as control or an shRNA targeting HUWE1. After selection cells were exposed to UVB. Lysates of non-treated cells were analyzed for HUWE1 knockdown (left panel). The right panel shows MCL1 immunoblots of UVB-treated samples with CDK2 as loading control.

B. HUWE1-mediated *in vivo* ubiquitination of MCL1. HeLa cells were transiently transfected with plasmids coding for MCL1, His-ubiquitin and HA-HUWE1 and treated with 4 μM MG-132 for 4 hrs. 36 hrs after transfection cells were lysed and His-ubiquitin-modified proteins were precipitated with Ni-NTA-Agarose. The eluates were probed with α-MCL1 antibody. Non-precipitated lysate served as loading control.

C. *In vivo* ubiquitination of MCL1 increases after UVB treatment. HeLa cells were exposed to 500 J/m^2 UVB and after indicated time points the ubiquitination analysis was performed as described in (B). Immunoblots of non-precipitated lysates were probed with an antibody recognizing phosphorylated CHK1 as control for UVB-induced DNA damage.

D. *In vivo* ubiquitination of MCL1 dependent on endogenous HUWE1. The experiment was carried out as described in (B) except that HUWE1-depleted HeLa cells are compared to non-depleted control cells.

E. *In vivo* ubiquitination of MCL1 after HUWE1 inhibition. HeLa cells were transfected with plasmids expressing MCL1, His-ubiquitin and HA-HUWE1. BI8622 was added at the indicated concentrations for 16 hrs. DMSO (D) served as control. Ubiquitin-modified proteins were pulled down by Ni-NTA-Agarose and immunoblots of the eluates were probed with an antibody against MCL1. Input control corresponds to 1 % of total lysate (upper panel). The lower panel shows mean of relative MCL1 ubiquitination +/- SD (n=5). The IC_{50} value was calculated using a four parameter logistic equation model.

Supplementary Figure S5: Characterization of HUWE1 inhibitors.

A. Ls174T cells were treated with the indicated concentrations of HUWE1 inhibitors or DMSO for 8 or 16 hrs. As positive control for DNA damage, Ls174T cells were exposed to 500 J/m^2 UVB and harvested 3 hrs later. Immunoblots of the lysates were incubated with indicated antibodies.

B. Colony assay of several colon carcinoma lines upon HUWE1 inhibition. The indicated cell lines were grown in the presence of HUWE1 inhibitors or DMSO as control. Cells were stained after five days. Inhibitors were used at 20 μM final concentration.

Supplementary Figure S6: IC_{50}-shift of BI8622 upon partial depletion of HUWE1.

A. RQ-PCR assays documenting reduction of HUWE1 mRNA levels in Ls174T cells upon shRNA-mediated depletion. Since a complete depletion of HUWE1 arrests proliferation, cells that grew out from an shRNA-depleted culture as described in Figure 1A were selected to achieve a partial depletion of HUWE1.

B. Crystal violet staining of HUWE1 inhibitor-treated Ls174T cells after HUWE1 depletion. Control or HUWE1-depleted cells were treated with indicated amounts of BI8622, DMSO served as solvent control. Five days later cells were stained with crystal violet (upper panel).

C. Quantification of the staining. Error bars show standard deviation of four independent replicates (n=4). IC_{50}-values were calculated using a four-parameter logistic equation model. p-value was determined by a two-tailed, heteroskedastic Student's t-test.

Supplementary Figure S7: Further characterization of BI8622 and BI8626.

A. Data from the growth curve shown in Figure 4C were combined with the FACS analysis shown in 4B to calculate the length of individual cell cycle phases (right panel). Data show mean +/- SD (n=3).

B. Cell cycle distribution of murine ES cells. ES cells were cultivated for 48 hrs in the presence of LIF and HUWE1 inhibitors. 1 h prior to harvest, cells were labeled with BrdU. Error bars show SD of technical triplicates from one representative experiment (n=2).

C. Stability of HUWE1 inhibitors in the presence of human liver microsomes. Microsomes were incubated with HUWE1 inhibitors BI8622 or BI8626 (10 µM) in the presence of NADPH for indicated time points. Extracted compounds were subjected to MS analysis. Error bars represent SD of three independent biological replicates (n=3). Half-life was calculated using non-linear curve fitting (one-phase decay).

D. Plasma levels of HUWE1 inhibitors after intraperitoneal injection. At the start of the experiment, HUWE1 inhibitors were injected (which would yield a concentration of 100µM assuming complete equilibration among all tissues). At the indicated times afterwards, mice were sacrificed and plasma levels determined by mass spectrometry.

Supplementary Figure S8: Effect of HUWE1 inhibition on p53.

Ls174T cells were stably infected with control vector or shRNA targeting p53. Cell pools were incubated with DMSO or HUWE1 inhibitors. The upper panel shows immunoblots documenting expression of p53, the lower panels shows RQ-PCR assays of the indicated MYC target genes. Error bars show SD of triplicate technical assays from one representative experiment (n=2).

Supplementary Figure S9: Effect of HUWE1 depletion or inhibition on the MYC/MAX/MXD/MNT network.

A. Immunoblots documenting MXD/MNT protein levels after depletion or inhibition of HUWE1. Ls174T cells were either infected with retroviruses expressing an shRNA targeting HUWE1 (left panel) or treated with BI8622 or BI8626 (20µM) (right panel). DMSO served as solvent control. Immunoblots of the lysates were analyzed for expression of MNT, MXD1, MXD3, MXD4 and Vinculin as loading control.

B. To determine whether HUWE1 inhibition affects complex formation of MYC with MAX, Ls174T cells were treated with BI8622 or BI8626 (20 µM), DMSO served as solvent control. Cell lysates were immuno-precipitated with MYC antibody or IgG as control and immunoblots of the eluates were probed with indicated antibodies. As input control 2 % of the lysate was loaded.

Supplementary Figure S10: Localization of MYC upon HUWE1 depletion or inhibition.

A. Immunofluorescence staining of MYC in HUWE1-depleted cells. Ls174T cells were infected retrovirally with an shRNA targeting HUWE1. After selection cells were plated on cover slips, fixed and stained with primary MYC antibody and AlexaFluor568-coupled secondary antibody. Hoechst dye was used to stain nuclei.

B. Immunofluorescence staining of MYC upon HUWE1 inhibition. Ls174T cells were treated with DMSO as control, BI8622 or BI8626 (20µM) and an immunofluorescence was carried out as described in (A).

Supplementary Figure S11: Characterization of HUWE1 effects on MIZ1 stability.

A, Effect of proteasome inhibition on HUWE1-induced downregulation of MIZ1. HeLa cells were transfected with plasmids encoding MIZ1 and HA-HUWE1. Cells were treated with BI8622 (20 µM) for 24 hrs, DMSO served as solvent control. MG132 was used at 10 µM for 6 hrs. Lysates were analyzed for expression of HUWE1 and MIZ1, Vinculin was used as loading control.

B. Immunoblots documenting levels of the indicated proteins in PAM212 keratinocytes stably infected with retroviruses expressing two different shRNAs against murine HUWE1 or a control vector.

C. Effects of HUWE1 inhibition on MYC and MIZ1 levels. PAM212 keratinocytes were treated with the indicated concentrations of HUWE1 inhibitors or DMSO as control. Cells were lysed and immunoblots probed with the indicated antibodies.

Supplementary Figure S12: ChIP-sequencing of MYC and MIZ1 upon HUWE1inhibition.

A. Table describing statistics of the performed ChIP-sequencing experiments. Peaks were called with MACS (Zhang et al, 2008), and filtered for false discovery rate (FDR) and for localization in promoters (-1kb to +0.5kb).

B. Examples of MIZ1 binding at individual core promoters after treatment with BI8622. Shown is an example for a direct MYC (E-box containing) target gene (*HSPE1*) as well as a direct target gene of MIZ1 (*VAMP4*).

Supplementary Figure S13: Characterization of HUWE1 effects on MYC and MIZ1 target genes.

A. Chromatin immunoprecipitation documenting binding of MYC and acetylation of histone H3 at the *HSPE1* and *ACTB* promoters in Ls174T cells upon HUWE1 depletion. Error bars show SD of technical triplicate assays from one representative experiment (n=2). p-values were calculated using Student's t-test (**p-value<0.001).

B. Chromatin immunoprecipitation documenting binding of MYC and acetylation of histones H3 and H4 at the indicated promoters in Ls174T cells upon HUWE1 inhibition. Ls174T cells were treated with HUWE1 inhibitors or DMSO as control. Error bars display SD from technical triplicate assays from one representative experiment (n=3). p-values were calculated using Student's t-test (*p-value<0.05; **p-value<0.001).

C. GSEA documenting expression of direct target genes of MIZ1 after HUWE1 depletion or treatment with HUWE1 inhibitors. MIZ1 binding data were taken from (Wolf et al, 2013).

Supplementary Table S1

shRNA	Sequence
shHUWE1-1	cccgcatgatcttgaattt
shHUWE1-2	gcagcagtacagactttaa
shHUWE1-3	gtgggaagctgctgatgta
shHUWE1-4	gcccaaccctcctcttata
shHUWE1ms-1	cacgaatgatcttgaattt
shHUWE1ms-2	ggtagatgtccttcaaata
shMIZ1	cctgtccaagcacatcatcat
shp53	ggtgaaccttagtacctaaa
sh scrambled	cataagctgagatacttca

Supplementary Table S2

Antibody	Clone/Origin
AcH3	Upstate (06-599)
AcH4	Upstate (06-866)
ARF-BP1	Abcam (ab65153)
CDK2	Santa Cruz (M2, sc-163)
H2B	Abcam (ab1790)
H3	Abcam (ab1791)
HA	Covance (16B12)
HDAC2	Santa Cruz (H-54, sc-899)
MXD1	Santa Cruz (C-19, sc-222)
MXD3	Santa Cruz (E-20, sc-933)
MXD4	B. Lüscher
MCL1	Santa Cruz (S-19, sc-19)
MIZ1	10E2 (group Eilers)
MNT	Santa Cruz (H-132, sc-769)
MYC	9E10

MYC	Santa Cruz (N-262, sc-764)
N-MYC	BD (B8.4.B)
Oct-4	Santa Cruz (H-134, sc-9081)
p53	Santa Cruz (DO-1, sc-126)
TopBP1	Novus (NB 100-217)
Vinculin	Sigma (hVIN-1)
β-tubulin	Chemicon (MAB3408)

Supplementary Table S3

	**Forward primer**	**Reverse primer**
**Primers for RQ-PCR**		
ACTB	cctcgcctttgccgatcc	ggatcttcatgaggtagtcagtc
CCNA2	tgtctcatggaccttcacca	ctctggtgggttgaggagag
CDC25A	tggataaaaatccaaacctaggag	caggtgcccctggttaga
CDKN1A	cgatgccaacctcctcaacga	tcgcagacctccagcatcca
GADD45A	cagaagaccgaaaggatgga	atctctgtcgtcgtcctcgt
HSPE1	catcatgttgatgccatttca	tggaggcaccaaagtagttct
HUWE1	ccagaagttcttcttgagggtact	gcctaaaccggaggaacc
MUC2	caagatcttcatggggagga	gaacacggtggtcctcttgt
MYC	caccagcagcgactctga	gatccagactctgaccttttgc
NCL	ccacttgtccgcttcaca	tcttggggtcaccttgattt
ODC1	aaagttggttttgcggattg	cgaaggtctcaggatcggta
RPS14	ggcagaccgagatgaatcctca	caggtccaggggtcttggtcc
TP53	ccgcagtcagatcctagcg	aatcatccattgcttgggacg
VAMP4	aaggagaaatcttttggaagatga	ggtccagatggtccccttag
B2MG	gtgctcgcgctactctctc	gtcaacttcaatgtcggat
**Primers for ChIP**		
ACTB	cgttccgaaagttgccttt	gccgctgggttttataggg
HSPE1	ctcggttccagaactttcca	ggtgaaagaactaccccttcg
Intergenic region (IR)	ttttctcacattgccccctgt	tcaatgctgtaccaggcaaa
VAMP4	cagtggttgttcctcccta	ccgagccctattcacctaaa

Figure S1

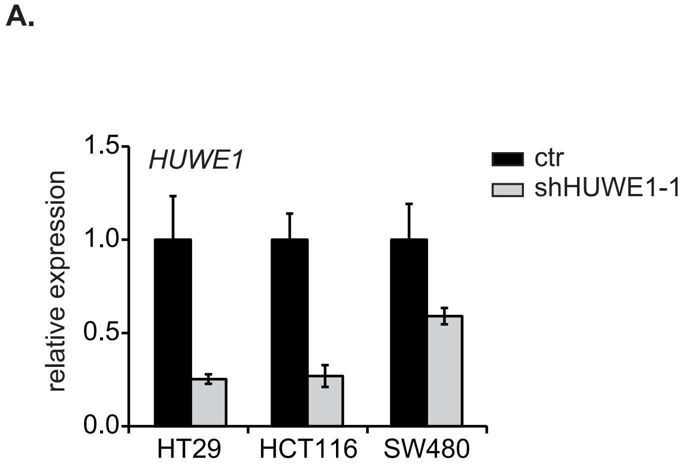
A.
HUWE1
relative expression
1.5
1.0
0.5
0.0
HT29
HCT116
SW480
ctr
shHUWE1-1

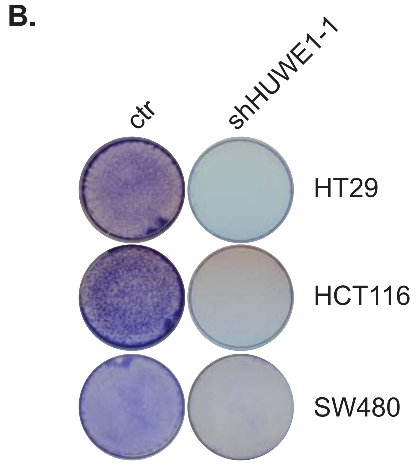
B.
ctr
shHUWE1-1
HT29
HCT116
SW480

Figure S2

A.

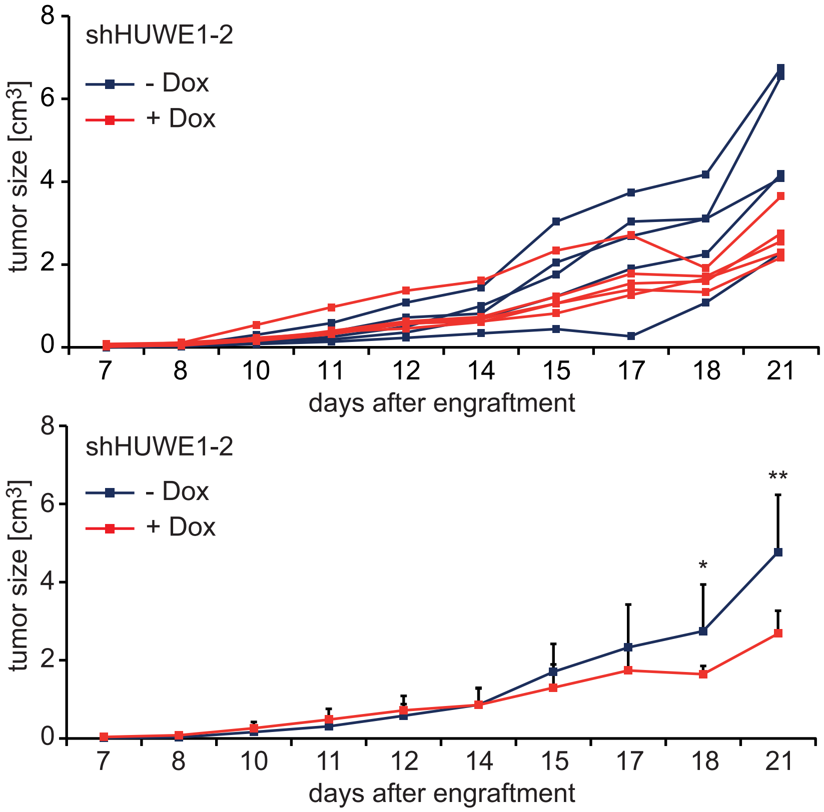

B.

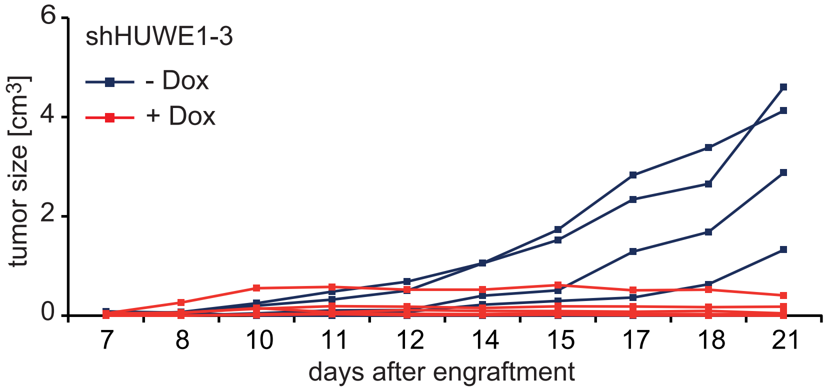

C.

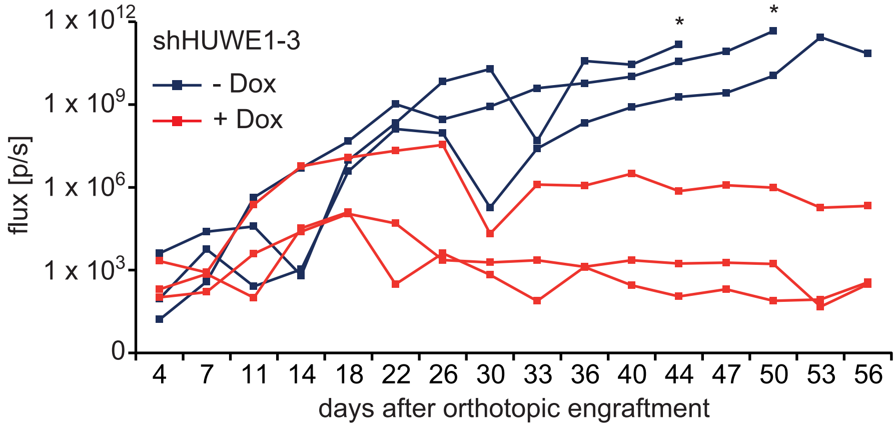

Figure S3

A.

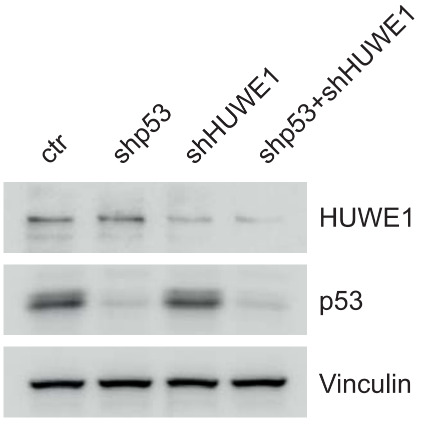
ctr
shp53
shHUWE1
shp53+shHUWE1
HUWE1
p53
Vinculin

B.

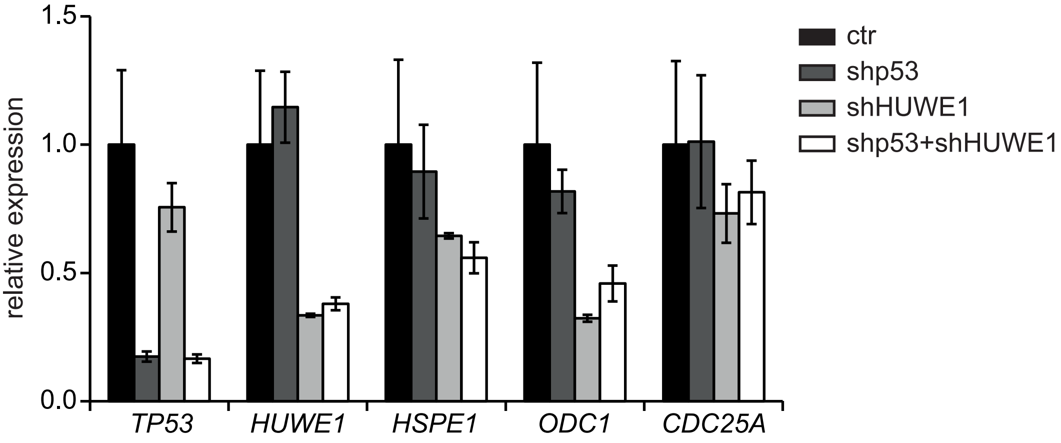
1.5
1.0
0.5
0.0
relative expression
TP53
HUWE1
HSPE1
ODC1
CDC25A
ctr
shp53
shHUWE1
shp53+shHUWE1

Figure S4

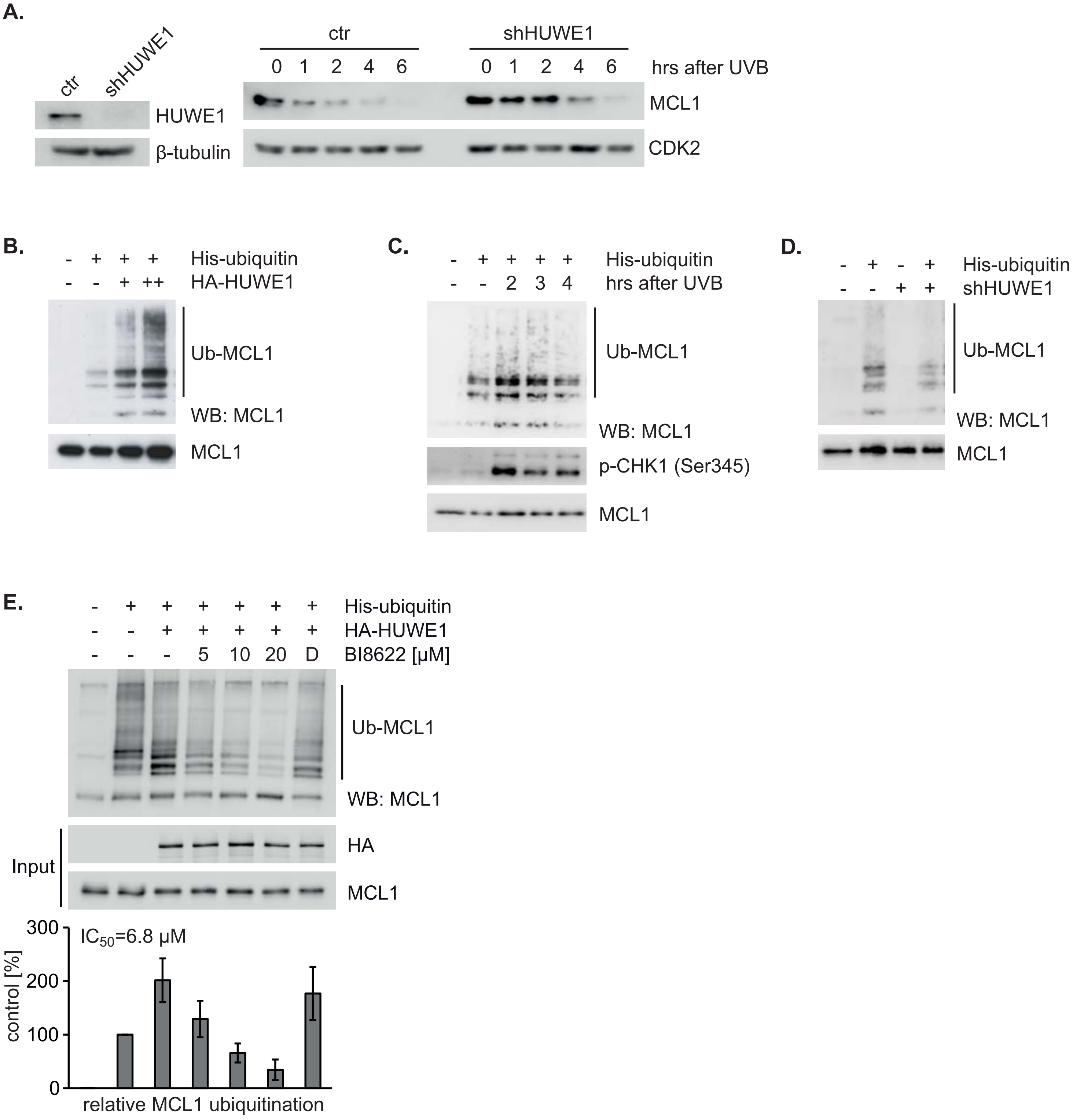
A.
ctr
shHUWE1
HUWE1
β-tubulin
ctr
shHUWE1
0 1 2 4 6
0 1 2 4 6
hrs after UVB
MCL1
CDK2
B.
His-ubiquitin
HA-HUWE1
Ub-MCL1
WB: MCL1
MCL1
C.
His-ubiquitin
hrs after UVB
Ub-MCL1
WB: MCL1
p-CHK1 (Ser345)
MCL1
D.
His-ubiquitin
shHUWE1
Ub-MCL1
WB: MCL1
MCL1
E.
His-ubiquitin
HA-HUWE1
BI8622 [μM]
5 10 20 D
Ub-MCL1
WB: MCL1
Input
HA
MCL1
IC50=6.8 μM
control [%]
300
200
100
0
relative MCL1 ubiquitination

Figure S5

A.

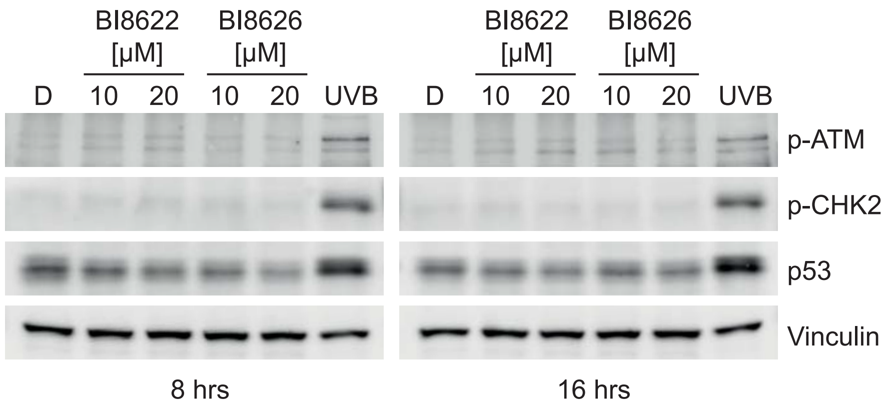
BI8622
[μM]
BI8626
[μM]
BI8622
[μM]
BI8626
[μM]
D
10
20
10
20
UVB
D
10
20
10
20
UVB
p-ATM
p-CHK2
p53
Vinculin
8 hrs
16 hrs

B.

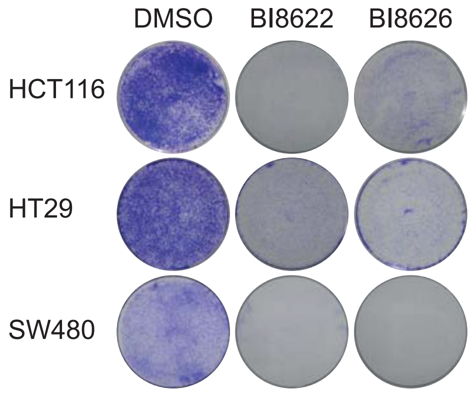
DMSO
BI8622
BI8626
HCT116
HT29
SW480

Figure S6

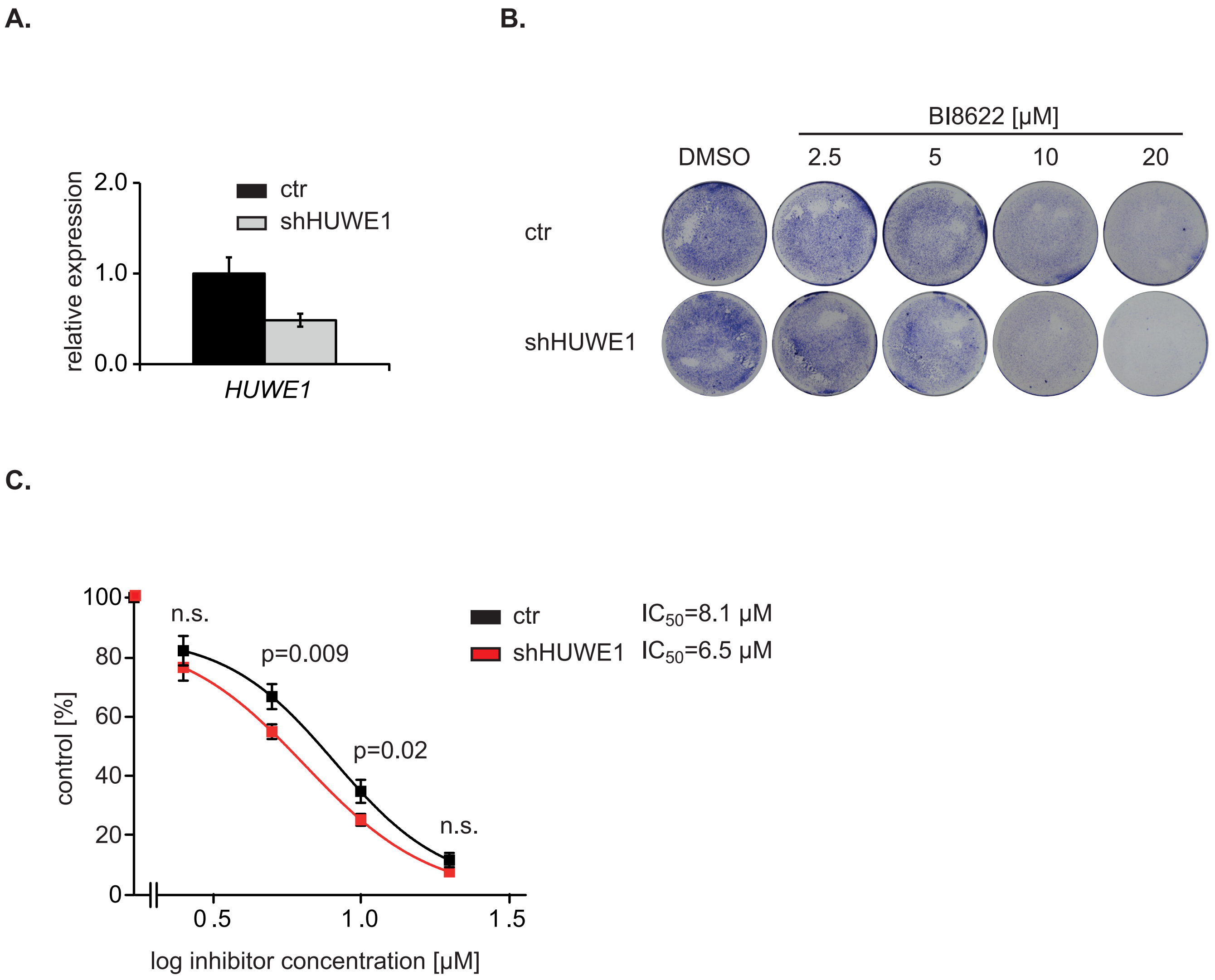
A.
B.
C.
relative expression
2.0
1.0
0.0
ctr
shHUWE1
HUWE1
BI8622 [µM]
DMSO
2.5
5
10
20
ctr
shHUWE1
100
80
60
40
20
0
control [%]
n.s.
p=0.009
p=0.02
n.s.
ctr
shHUWE1
IC50=8.1 µM
IC50=6.5 µM
0.5
1.0
1.5
log inhibitor concentration [µM]

Figure S7

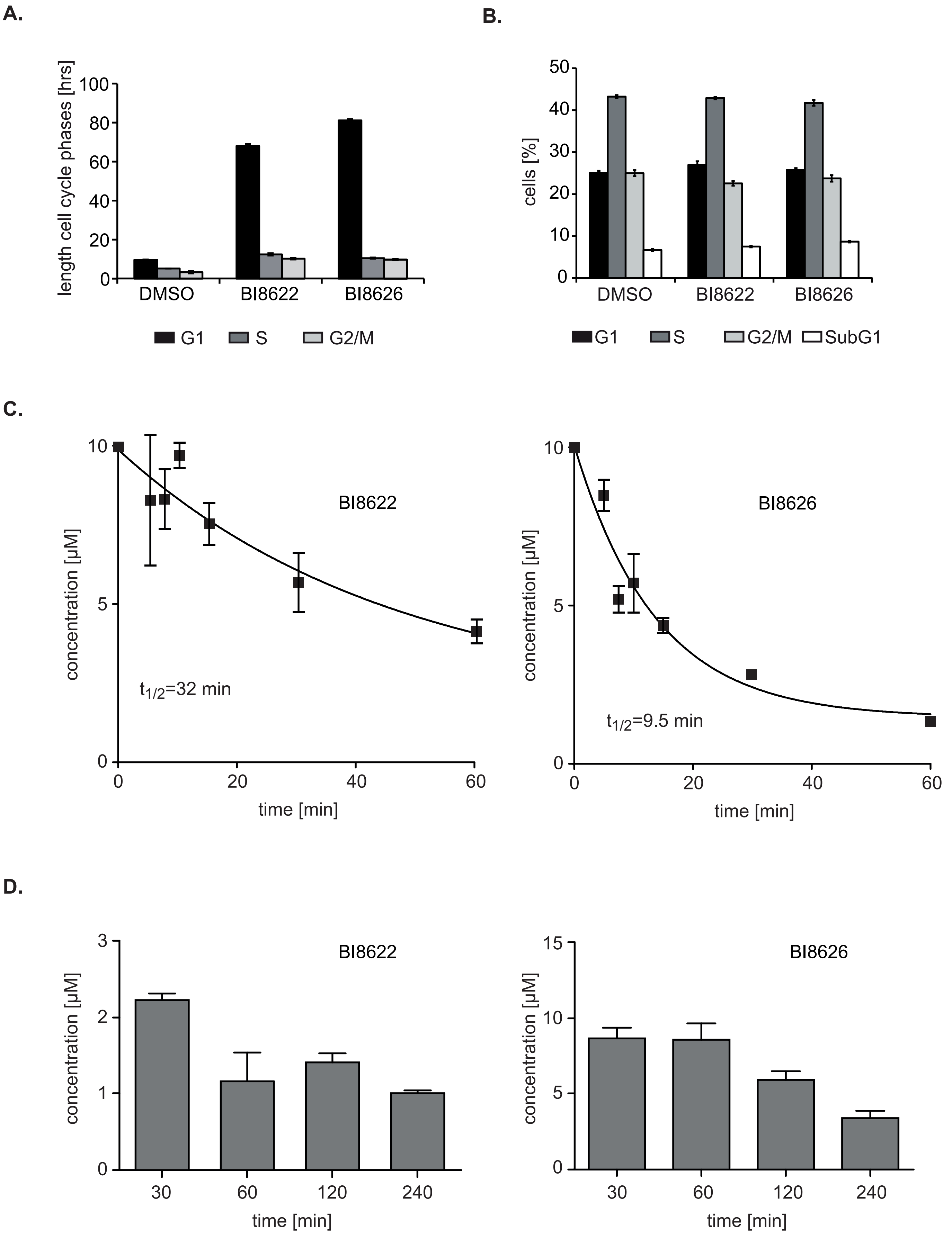
A.
length cell cycle phases [hrs]
100
80
60
40
20
0
DMSO
BI8622
BI8626
G1
S
G2/M
B.
cells [%]
50
40
30
20
10
0
DMSO
BI8622
BI8626
G1
S
G2/M
SubG1
C.
concentration [µM]
10
5
0
BI8622
t1/2=32 min
0
20
40
60
time [min]
concentration [µM]
10
5
0
BI8626
t1/2=9.5 min
0
20
40
60
time [min]
D.
concentration [µM]
3
2
1
0
BI8622
30
60
120
240
time [min]
concentration [µM]
15
10
5
0
BI8626
30
60
120
240
time [min]

Figure S8

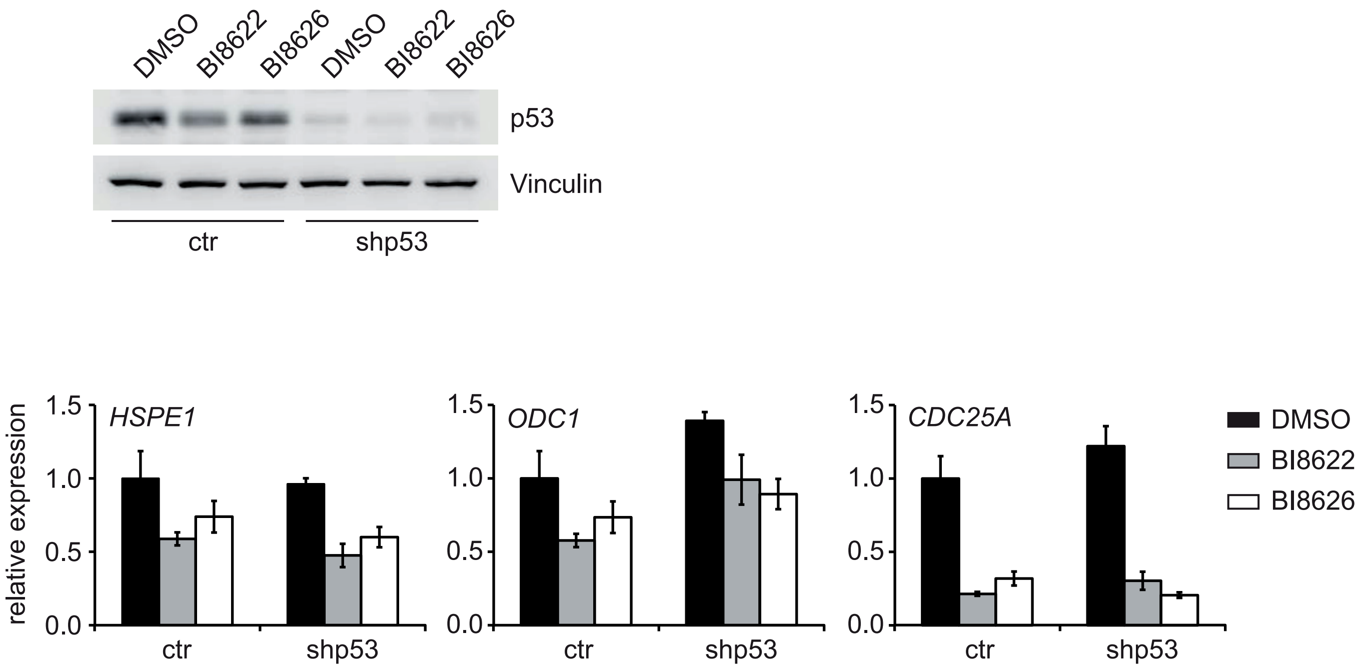
DMSO
BI8622
BI8626
DMSO
BI8622
BI8626
p53
Vinculin
ctr
shp53
relative expression
HSPE1
ODC1
CDC25A
1.5
1.0
0.5
0.0
ctr
shp53
DMSO
BI8622
BI8626

Figure S9

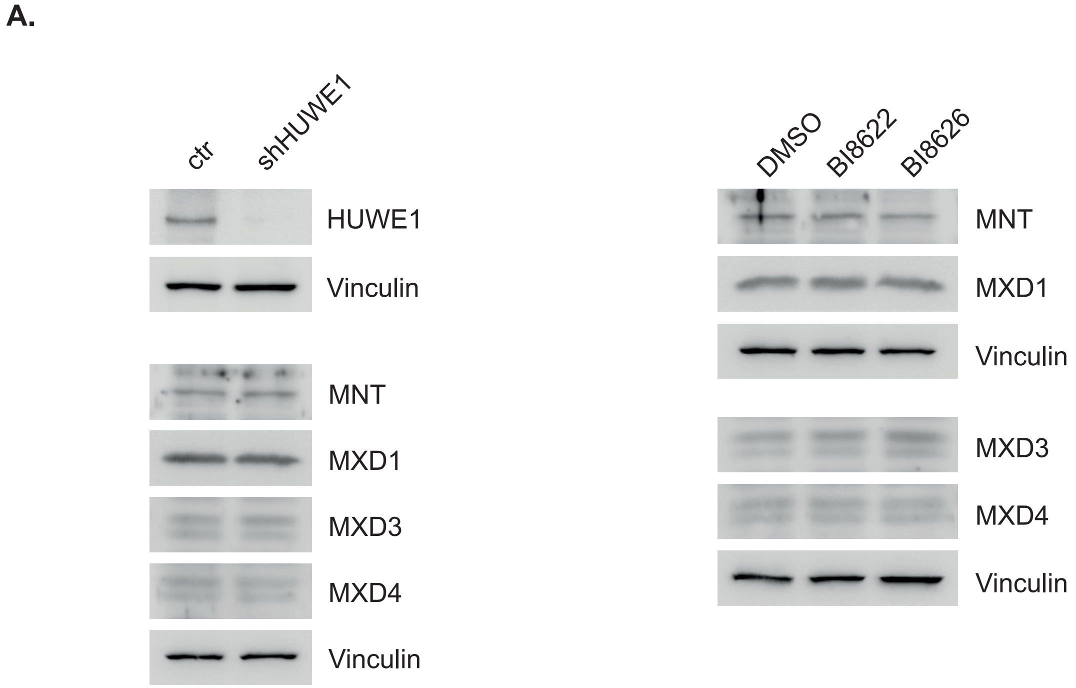
A.
ctr
shHUWE1
HUWE1
Vinculin
MNT
MXD1
MXD3
MXD4
Vinculin
DMSO
BI8622
BI8626
MNT
MXD1
Vinculin
MXD3
MXD4
Vinculin

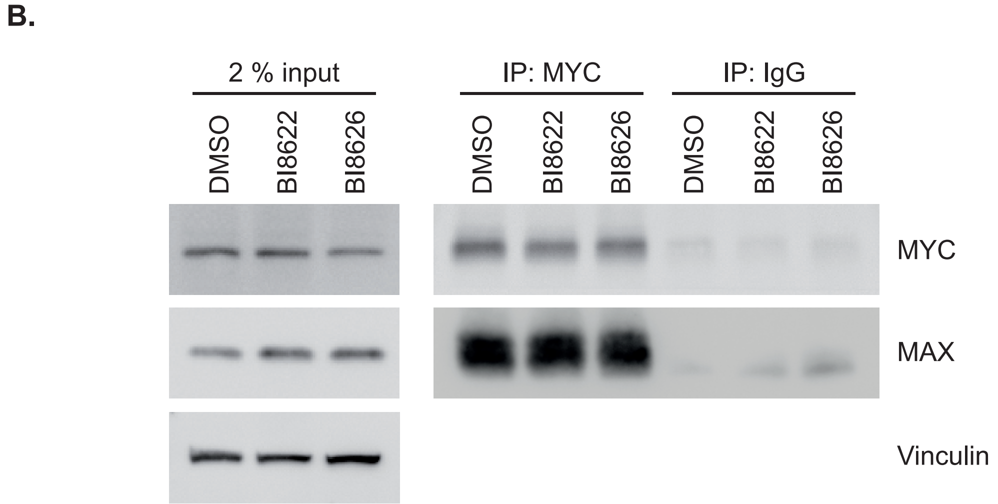
B.
2 % input
IP: MYC
IP: IgG
DMSO
BI8622
BI8626
DMSO
BI8622
BI8626
DMSO
BI8622
BI8626
MYC
MAX
Vinculin

Figure S10

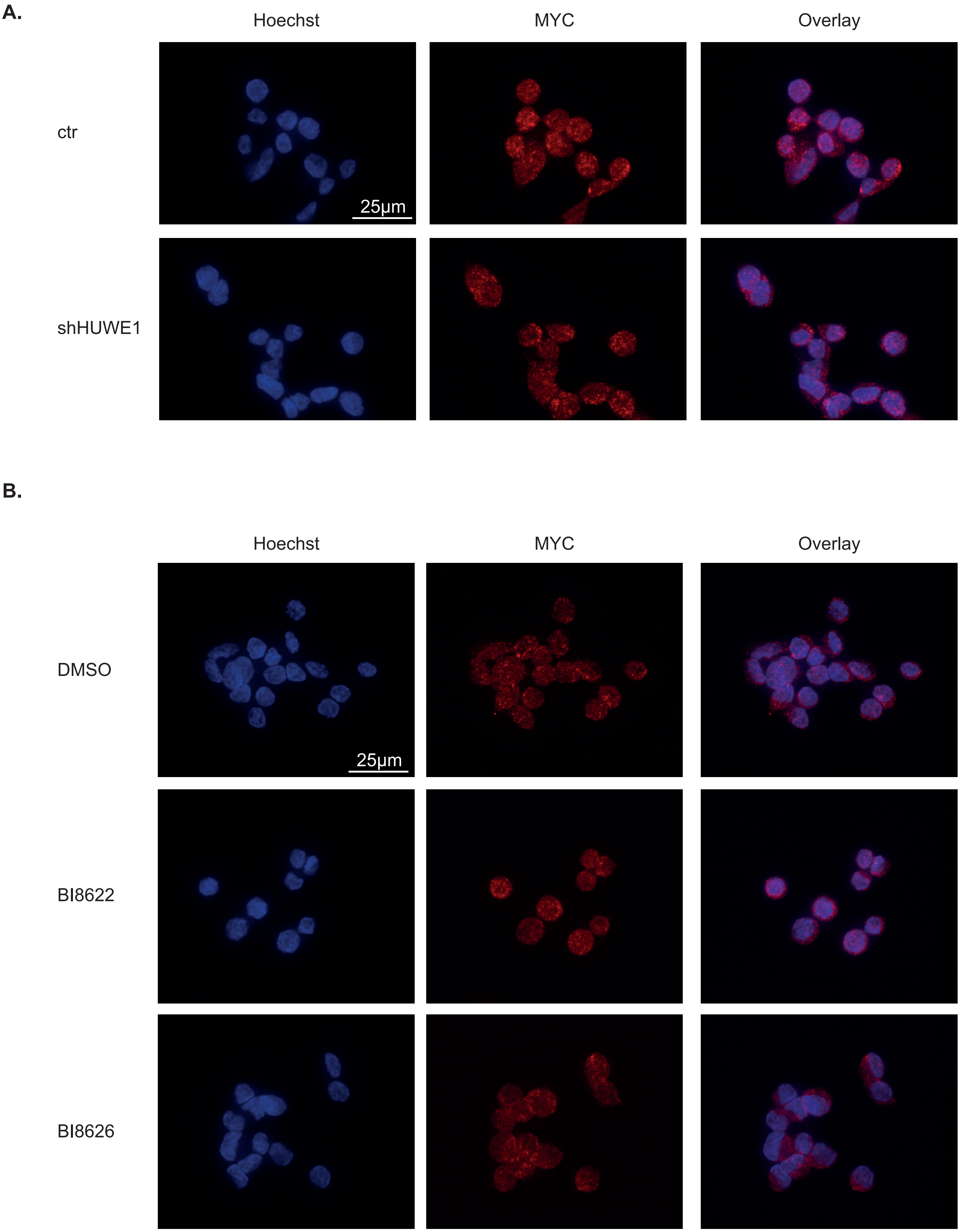
A.
Hoechst
MYC
Overlay
ctr
shHUWE1
25µm
B.
Hoechst
MYC
Overlay
DMSO
BI8622
BI8626
25µm

Figure S11

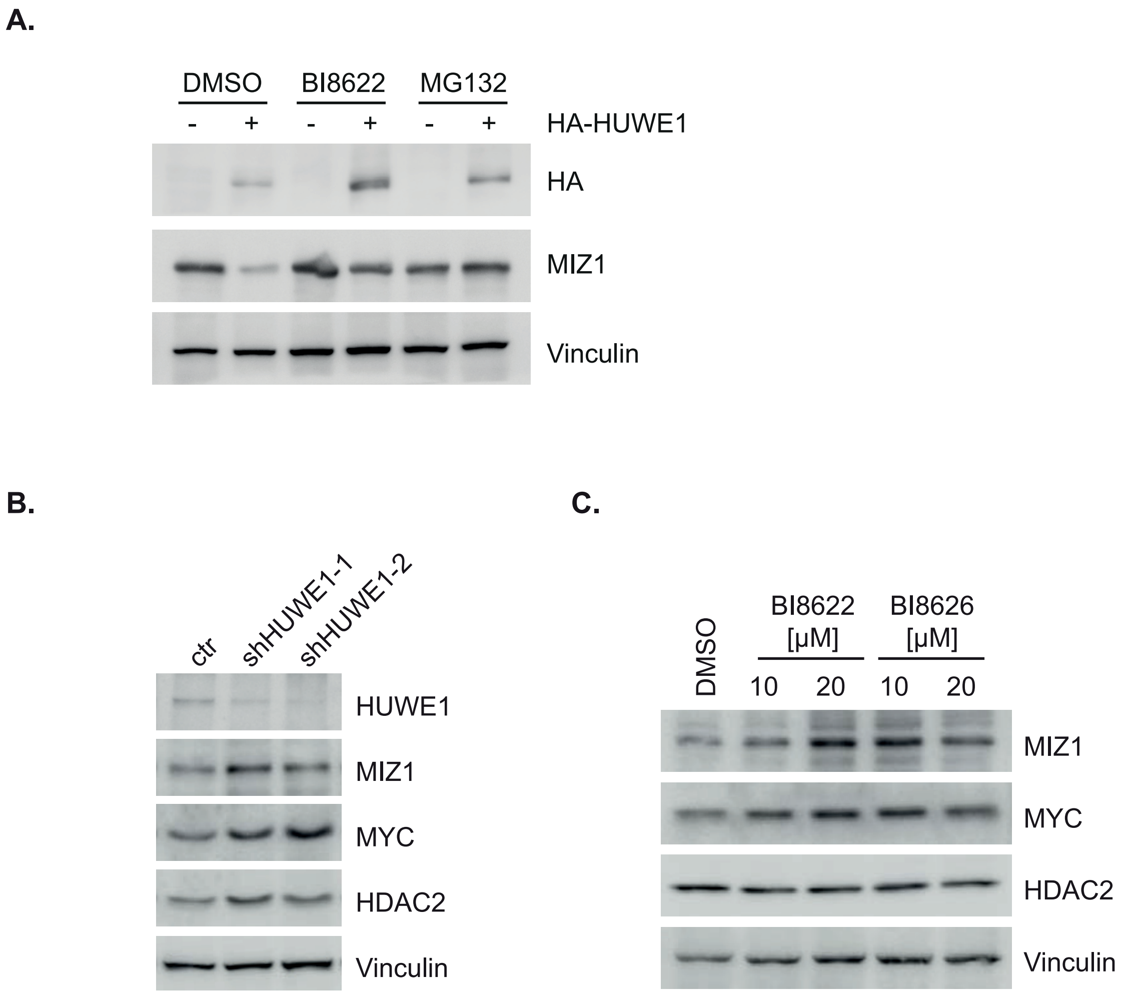

A.
DMSO
BI8622
MG132
- + - + - +
HA-HUWE1
HA
MIZ1
Vinculin
B.
ctr
shHUWE1-1
shHUWE1-2
HUWE1
MIZ1
MYC
HDAC2
Vinculin
C.
DMSO
BI8622 [μM]
BI8626 [μM]
10 20 10 20
MIZ1
MYC
HDAC2
Vinculin

Figure S12

A.

	mapped reads		promotor-proximal binding sites (FDR 0.1)	
	DMSO	BI8622	DMSO	BI8622
MYC	10,728,078	10,710,164	548	1,483
MIZ1	10,698,781	10,682,658	1,733	5,746
IgG	10,703,678	10,680,730		

B.

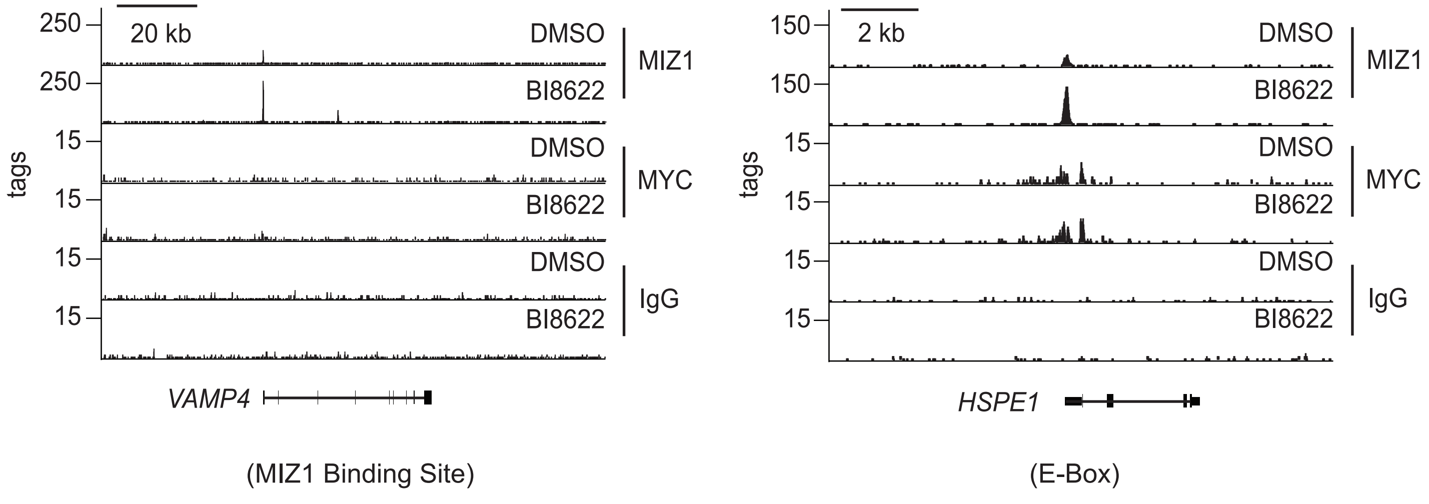

Figure S13

A.

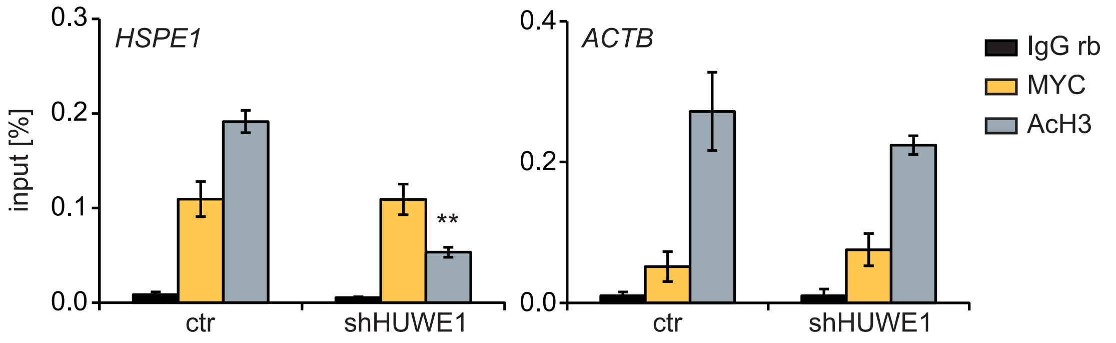

B.

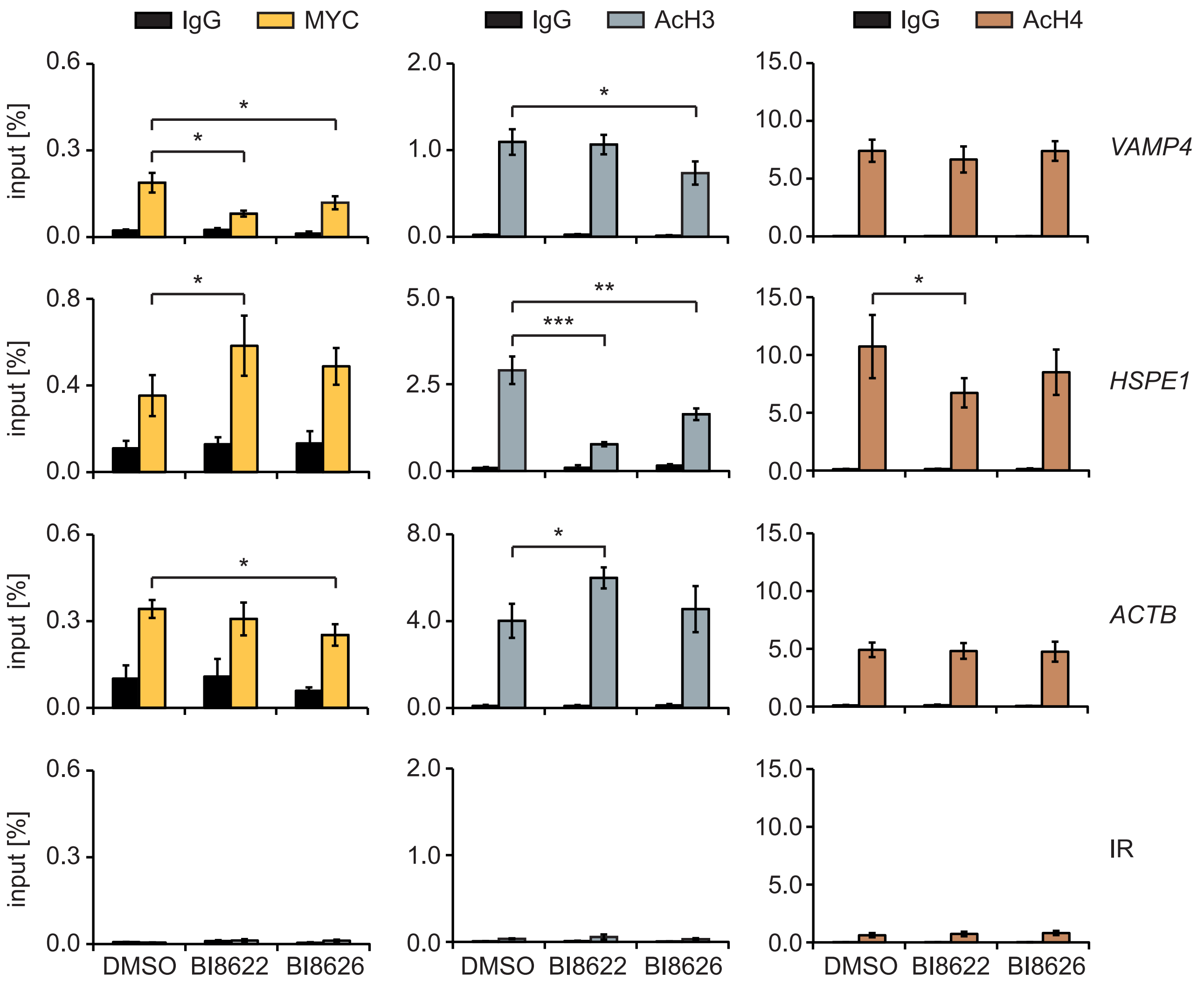

C.

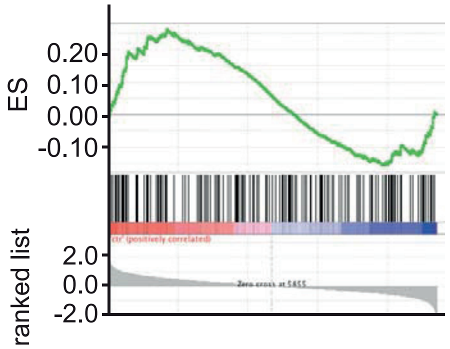

MYC/MIZ1-bound
promoters and MIZ1 motif

NES 1.20
q-value 0.45
p-value 0.12
